# BUILDING BRIDGES ACROSS THE GENERATIONS: RESEARCH ON DIVERSE APPROACHES FOR INTERGENERATIONAL CONNECTIONS

**DOI:** 10.1093/geroni/igad104.1443

**Published:** 2023-12-21

**Authors:** Amy Eisenstein, Ernest Gonzales

## Abstract

Intergenerational bonds are a unique and critically important set of social connections that have powerful impacts on families and communities, as well as the older and younger people themselves. America is increasingly age-segregated, so that naturally occurring, cross-generational contacts are not as common as they have been in previous eras. Research from the FrameWorks Institute and others suggest that ageist language can further divide the generations, create a sense of “othering,” and contribute to the isolation and minimization of older adults. Conversely, studies also show that meaningful intergenerational programming, which brings to light the strengths of both generations, may reduce social isolation and loneliness and enhance the physical and mental well-being of older adults. This symposium will bring together three research projects each exploring diverse approaches to intergenerational connections. The first paper will present lessons learned from a study of digital communication between grandparents and grandchildren during the COVID-19 pandemic, and the implications this shift in communications has on the psychological well-being of older grandparents. The second paper will present findings regarding the physical and psychosocial outcomes experienced by older adults who provide intergenerational tutoring in elementary school settings. The final session will highlight the process of integrating implementation science frameworks into a multistage study design aimed to assess the implementation of an intergenerational technology program within higher education. The session will wrap-up with a discussion on how these projects fit within the broader scope of intergenerational programs, and will highlight gaps and further opportunities for research in this field.

